# Supplementary material for: Characterizing superspreading events and age-specific infectiousness of SARS-CoV-2 transmission in Georgia, USA
Source: Proc Natl Acad Sci U S A. 2020 Aug 20;117(36):22430–5. doi: 10.1073/pnas.2011802117 (PMC7486752; doi:10.1073/pnas.2011802117)
Supplement: Supplementary File [file pnas.2011802117.sapp.pdf]

Supplementary Information (SI)

Max SY Lau[a,1], **Bryan Grenfell**[b,c], **Michael Thomas**[d], **Michael Bryan**[d], **Kristin Nelson**[e], **and Ben Lopman**[e]

[a]Department of Biostatistics and Bioinformatics, Rollins School of Public Health, Emory University; [b]Department of Ecology and Evolutionary Biology, Princeton University, Princeton; [c]Fogarty International Center, National Institute of Health, USA; [d]Georgia Department of Public Health; [e]Department of Epidemiology, Rollins School of Public Health, Emory University

1. SI Text

Probability of Infection. We consider a Susceptible-Exposed-Infectious-Hospitalized/Recovered stochastic spatio-temporal model for individual patients. Let ψ_j denote the source of infection for an infected individual j, and let $G_j = (r_j, \theta_j)$ denote the distance r_j and angle θ_j measured from ψ_j to j.

Assuming complete observations, the probability of j getting infected at time E_j and position G_j will be

$$P(j, \psi_j) \times g(G_j; \eta, \hat{\boldsymbol{s}}) \times (1/r_j), \quad [1]$$

where

$$P(j, \psi_j) = \begin{cases} \alpha, & \text{if individual } j \text{ is a background infection,} \\ \beta(E_j), & \text{if } \psi_j \text{ is infectious at time } E_j. \end{cases} \quad [2]$$

Following [9], we consider

$$g(G_j; \eta, \hat{\boldsymbol{s}}) = f(r_j; \eta) \times h(\theta_j | r_j, \hat{\boldsymbol{s}}), \quad [3]$$

where $f(.)$ is the density function of an Exponential distribution parameterized by the mean η (generally, $f(.)$ is a monotonically decreasing density function that characterizes the likelihood over spatial spread over a distance). The function $h(.)$ takes into account potential heterogeneous mixing contributed by varying population densities across a study area. A natural approach in specifying $h(\theta_j | r_j, \hat{\boldsymbol{s}})$ is to use the population density along the circumference of the circle centered at the source infection ψ_j with the radius r_j, denoted by $\sigma(l | r_j, \hat{\boldsymbol{s}})$, to account for the effect of heterogeneous landscape, so that

$$\int_0^{\theta'} h(\theta | r, \hat{\boldsymbol{s}}) d\theta = \int_0^{l'} \sigma(l | r, \hat{\boldsymbol{s}}) dl, \quad [4]$$

where l' is the arc length corresponding to an arbitrary angle θ'. The incorporation of $h(.)$ has shown to be important for more realistically representing the spatial spread of Ebola outbreaks, more details are referred to [9]. After the day of intervention (i.e. issue of shelter-in-place order) the mean of the movement distance (i.e. η) is assumed to change by a proportion $q \times \eta$, where q is estimated empirically from the change of average movement distance computed from the Facebook mobility data (see main text).

Statistical Inference and Data-augmentation. We conduct Bayesian inference of the partially observed outbreak using the process of data augmentation supported by Markov chain Monte Carlo methods [8, 9, 24]. Let $\boldsymbol{\Theta} = (\alpha, \beta_1, \beta_2, \mu, c, \eta, \omega)$ (β_i is the baseline infectivity in age group i). Given observed partial data $\boldsymbol{y}$, the inference involves sampling from the joint posterior distribution $\pi(\boldsymbol{\Theta}, \boldsymbol{z} | \boldsymbol{y}) \propto L(\boldsymbol{\Theta}; \boldsymbol{z}) \pi(\boldsymbol{\Theta})$, where $\boldsymbol{z}$ represents the complete data and $\pi(\boldsymbol{\Theta})$ represents the prior distribution of model quantities, such that the complete $\boldsymbol{z}$ is reconstructed, or 'imputed'.

Parameters in $\boldsymbol{\Theta}$ are updated sequentially with a standard random-walk Metropolis-Hastings algorithm [24]. For example, a new parameter value α' is proposed from a normal distribution centered on the current value of α

$$\alpha' \sim N(0, \rho^2) \quad [5]$$

where ρ controls the step-size of the random-walk. β_1 and β_2 are highly correlated and to improve mixing, we use the Metropolis-Hastings algorithm with blockings for these two parameters. They are drawn using multivariate Normal distribution as proposal distributions, with the mean values taken to be current parameter values (i.e. random walk), and with entries in the covariance matrix estimated from the samples generated from a preliminary run of the unblocked version of the MCMC algorithm (i.e. each parameter is sampled sequentially using random-walk Metropolis-Hastings) [10]. The unobserved infection time E_j and the source of infections ψ_j and any missing symptom onset dates are also inferred following [8, 9, 23]. Denote ω_ψ as the set of eligible candidates for a new source of infection $\psi_j^{'}$ for j (i.e. ω_ψ contains a set of cases whose are infectious at E_j). We propose a new infecting source $i \in \omega_\psi$ to be $\psi_j^{'}$ with probability

$$p_{ij} \propto \beta_i f(r_j; \eta). \quad [6]$$

Note that the background infection can be accommodated by adding a permanent infectious source presenting an additional challenge of strength α to individual j. A newly proposed source is accepted or rejected depending on the M-H acceptance probability [23]. We use non-infomative uniform priors $U(0, 100)$.

2. SI Tables

DRAFT

Table S1. Posterior means of model parameters and their 95% C.I.

County	α	η	β_1	β_2	ω	μ	c
Cobb	0.02 [0.01,0.05]	0.12 [0.11,0.13]	0.14 [0.13,0.16]	0.05 [0.05,0.06]	0.07 [0.06,0.08]	9.43 [9.02,9.96]	6.372 [5.75,7.09]
DeKalb	0.09 [0.06,0.18]	0.18 [0.14,0.19]	0.09 [0.08,0.12]	0.04 [0.03,0.052]	0.06 [0.05,0.07]	11.24 [10.72,11.89]	6.69 [5.9,7.55]
Fulton	0.10 [0.07,0.19]	0.1 [0.09,0.11]	0.07 [0.06,0.08]	0.024 [0.02,0.031]	0.07 [0.06,0.09]	10.82 [10.42,11.50]	6.7 [6.06,7.38]
Gwinnett	0.08 [0.05,0.15]	0.16 [0.142,0.17]	0.062 [0.05,0.09]	0.03 [0.021,0.04]	0.04 [0.03,0.05]	9.5 [9.0,10.0]	4.71 [3.94,5.54]
Dougherty	0.03 [0.01,0.06]	0.19 [0.18,0.21]	0.21 [0.19,0.24]	0.055 [0.049,0.061]	0.17 [0.15,0.19]	8.7 [8.2,9.2]	4.8 [4.29,5.36]

DRAFT
